# TRPV1, but not TRPA1, in primary sensory neurons contributes to cutaneous incision-mediated hypersensitivity

**DOI:** 10.1186/1744-8069-9-9

**Published:** 2013-03-04

**Authors:** Marie E Barabas, Cheryl L Stucky

**Affiliations:** 1 Department of Cell Biology, Neurobiology and Anatomy, Medical College of Wisconsin, 8701 Watertown Plank Road, Milwaukee, WI 53226-0509, USA

**Keywords:** TRPV1, TRPA1, Postoperative pain, Sensory neuron, Keratinocyte, Mechanical, Heat, Skin, Cutaneous, Calcium imaging

## Abstract

**Background:**

Mechanisms underlying postoperative pain remain poorly understood. In rodents, skin-only incisions induce mechanical and heat hypersensitivity similar to levels observed with skin plus deep incisions. Therefore, cutaneous injury might drive the majority of postoperative pain. TRPA1 and TRPV1 channels are known to mediate inflammatory and nerve injury pain, making them key targets for pain therapeutics. These channels are also expressed extensively in cutaneous nerve fibers. Therefore, we investigated whether TRPA1 and TRPV1 contribute to mechanical and heat hypersensitivity following skin-only surgical incision.

**Results:**

Behavioral responses to mechanical and heat stimulation were compared between skin-incised and uninjured, sham control groups. Elevated mechanical responsiveness occurred 1 day post skin-incision regardless of genetic ablation or pharmacological inhibition of TRPA1. To determine whether functional changes in TRPA1 occur at the level of sensory neuron somata, we evaluated cytoplasmic calcium changes in sensory neurons isolated from ipsilateral lumbar 3–5 DRGs of skin-only incised and sham wild type (WT) mice during stimulation with the TRPA1 agonist cinnamaldehyde. There were no changes in the percentage of neurons responding to cinnamaldehyde or in their response amplitudes. Likewise, the subpopulation of DRG somata retrogradely labeled specifically from the incised region of the plantar hind paw showed no functional up-regulation of TRPA1 after skin-only incision. Next, we conducted behavior tests for heat sensitivity and found that heat hypersensitivity peaked at day 1 post skin-only incision. Skin incision-induced heat hypersensitivity was significantly decreased in TRPV1-deficient mice. In addition, we conducted calcium imaging with the TRPV1 agonist capsaicin. DRG neurons from WT mice exhibited sensitization to TRPV1 activation, as more neurons (66%) from skin-incised mice responded to capsaicin compared to controls (46%), and the sensitization occurred specifically in isolectin B4 (IB4)-positive neurons where 80% of incised neurons responded to capsaicin compared to just 44% of controls.

**Conclusions:**

Our data suggest that enhanced TRPA1 function does not mediate the mechanical hypersensitivity that follows skin-only surgical incision. However, the heat hypersensitivity is dependent on TRPV1, and functional up-regulation of TRPV1 in IB4-binding DRG neurons may mediate the heat hypersensitivity after skin incision injury.

## Background

Postoperative pain remains a major challenge to treatment following surgical procedures [1]. Pain management strategies for postoperative pain often require the use of opioids, which have a multitude of adverse side effects including nausea, constipation, tolerance and addiction [1,2]. Further, studies have shown that the use of opioids can increase the risk of developing chronic pain as they can exacerbate hyperalgesia in certain individuals [2,3]. Therefore, investigation into the mechanisms behind postoperative pain might identify novel pain therapeutic targets. Surgical incisions typically involve multi-tissue injury, including skin, muscle, fascia, peripheral nerves and vasculature, which adds to the complexity of understanding the underlying mechanisms of postoperative pain. Different subpopulations of sensory neurons innervate particular peripheral tissues [4,5], and pain mechanisms may vary depending on the types of tissue damaged during surgery. Therefore, discerning the contribution of different tissues to the generation and maintenance of postoperative pain might enable development of more targeted treatment strategies with fewer side effects.

The development of a mouse model of postoperative pain, adapted to the mouse from the previously defined rat model, has allowed combining the versatility of mouse transgenic lines with the ability to test rodent nociceptive behavior and nerve function [6,7]. The original model involved an incision through skin, fascia and muscle (skin plus deep), but a more recent model consists of just skin incision (skin-only) [6,7]. When comparing pain behaviors in the two models, both injury models elicit hypersensitivity to evoked mechanical and heat stimuli to a similar magnitude, although spontaneous/unprovoked pain behaviors occur only in the skin plus deep incision model [8,9]. These data suggest that skin-only incision injury alone is capable of sensitizing sensory nerve terminals to mechanical and heat stimuli in the postoperative pain model.

Studies have shown that Aδ and C fiber afferents innervating glabrous skin are sensitized to mechanical and heat stimuli after skin plus deep incision [10-12]. The Transient Receptor Potential Vanilloid 1 (TRPV1) channel was found to mediate the heat hypersensitivity following skin plus deep incision injury [13-15]. Here we investigated whether the heat hypersensitivity following skin-only incision injury is dependent on TRPV1. Several studies have shown that mechanical hypersensitivity develops independently of the TRPV1-mediated heat hypersensitivity [13-15]. Therefore, we investigated a non-TRPV1 mechanism that may mediate the incision-induced mechanical hypersensitivity.

The Transient Receptor Potential Ankyrin 1 (TRPA1) channel is another member of the TRP family and has been shown to mediate inflammation- and nerve injury-induced behavioral mechanical hyperalgesia [16-19]. TRPA1 is expressed on both Aδ and C fibers and also on keratinocytes in the epidermis [16-20]. We have recently shown that TRPA1 mediates the mechanical sensitization of a select population of cold- and mechano-sensitive C fibers [21]. Wei and colleagues [22] found that TRPA1 contributes to both mechanical hypersensitivity and non-evoked nocifensive behaviors following skin plus deep incision injury using a pharmacological approach in rats. However, use of this model prevents identification of the specific tissue in which TRPA1 mediates the afferent sensitization and nocifensive behavior. Therefore, we tested the hypothesis that TRPA1 contributes to mechanical hypersensitivity and TRPV1 contributes to heat hypersensitivity following skin-only incision injury. We used TRPA1-deficient (TRPA1 KO) [23] and TRPV1-deficient (TRPV1 KO) [24] mouse lines as well as a TRPA1 antagonist HC-030031 [25]. Further, we used calcium imaging to examine whether the TRPA1 and TRPV1 channels are functionally up-regulated in dorsal root ganglia (DRG) sensory neurons that project to the incised or corresponding sham region of the plantar hind paw. Our findings indicate that TRPA1 does not mediate the mechanical hypersensitivity and is not functionally up-regulated following skin-only incision. We also found that the heat hypersensitivity is dependent on TRPV1, and the TRPV1 channel is functionally up-regulated specifically in IB4-positive small-diameter neurons.

## Results

### Genetic ablation or acute pharmacological blockade of TRPA1 does not affect mechanical hypersensitivity following skin-only incision injury

**Figure 1:**
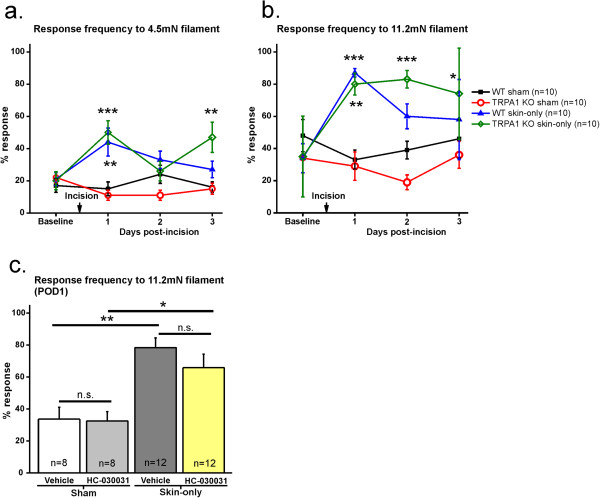
**Mechanical hypersensitivity is prevalent after skin incision with either genetic knockout or pharamacological block of TRPA1. a**. Response frequency to 4.5mN filament for WT and TRPA1 KO skin incised and sham mice. Measurements are reported as percent responses (out of 10 total applications of force). Both WT and TRPA1 KO mice exhibit increased mechanical sensitivity on postoperative day 1 (POD1) compared to sham controls (p = 0.0002; **p < 0.01; ***p < 0.0001; represents difference between skin-only and corresponding sham). There is no difference between WT and TRPA1 KO skin-only incised mice at any time point. 10 mice per group. **b**. Response frequency to 11.2mN filament for WT and TRPA1 KO skin incised and sham mice. Both WT and TRPA1 KO mice exhibit increased mechanical sensitivity at POD1 as compared to sham controls (p < 0.0001; **p < 0.001; ***p < 0.0001; *p < 0.05; represents difference between skin-only and corresponding sham). There is no difference between WT and TRPA1 KO skin-only incised mice at any time point after skin incision. These mice are the same as those used in Figure 1a. **c**. Response frequencies to 11.2mN filament on POD1 after skin-only incision or sham with treatment with vehicle or TRPA1 antagonist, HC-030031. Skin-only incised mice treated with vehicle as well as those treated with HC-030031 exhibit significantly more responses to the mechanical force than the sham groups (p < 0.0001; **p < 0.001; *p < 0.05). 8 mice per sham group; 12 mice per skin-only incision group.

In order to determine whether TRPA1 mediates the mechanical hypersensitivity induced by cutaneous injury, we conducted skin-only incision or sham procedures on wild-type (WT) and TRPA1-deficient “knockout” (TRPA1 KO) [23] mice. Sham procedures consisted of exposing animals to the same duration and dose of anesthetic but no incision. Mechanical sensitivity was calculated as the percentage of paw withdrawal responses out of total applications of a von Frey monofilament [26], and mechanical hypersensitivity was defined as an increase in mechanical responsiveness compared to sham mice. We chose 2 monofilaments: one filament that elicited an approximate 20% response rate at baseline (4.5mN force; Figure 1a) and a second filament that elicited an approximate 40% response rate at baseline (11.2mN force; Figure 1b). There was no difference in baseline mechanical sensitivity between WT and TRPA1 KO mice (Figure 1a, b). On post-operative day 1 (POD1), WT and TRPA1 KO mice both exhibited significantly increased mechanical sensitivity compared to their respective baselines and sham controls (Figure 1a, b). There was no difference in mechanical responsiveness between skin-only incised WT and TRPA1 KO mice. These data suggest that genetic ablation of TRPA1 does not affect mechanical hypersensitivity following skin-only incision injury.

Potential compensatory mechanisms in the TRPA1 KO mice could complicate our interpretation of the behavior data using this mouse line. Therefore, we conducted behavior on sham and skin-only incised WT mice after intraplantar (local) injection of a TRPA1 antagonist, HC-030031 (100 μg/site), a concentration demonstrated to inhibit mechanical hypersensitivity with hindpaw inflammation in mice [25]. Control mice were injected with vehicle. For this experiment, we measured the percentage of responses to the 11.2 mN filament on POD1 since the skin-only incision had the greatest effect on mechanical responsiveness with those parameters. Both vehicle- and HC-030031-treated incised mice exhibited significantly more responses than their corresponding sham controls (Figure 1c). The response frequencies between incised mice treated with vehicle and those treated with HC-030031 were not different. Since we did not observe an effect of HC-030031 in either incised or sham mice, it appears that off-target effects on mechanical sensitivity were minimal. Taken together, these data suggest that TRPA1 does not mediate mechanical hypersensitivity caused by skin injury in mice.

### Genetic ablation or acute pharmacological blockade of TRPA1 does not affect mechanical hypersensitivity or guarding behavior following cutaneous and muscle surgical injury in mice

**Figure 2:**
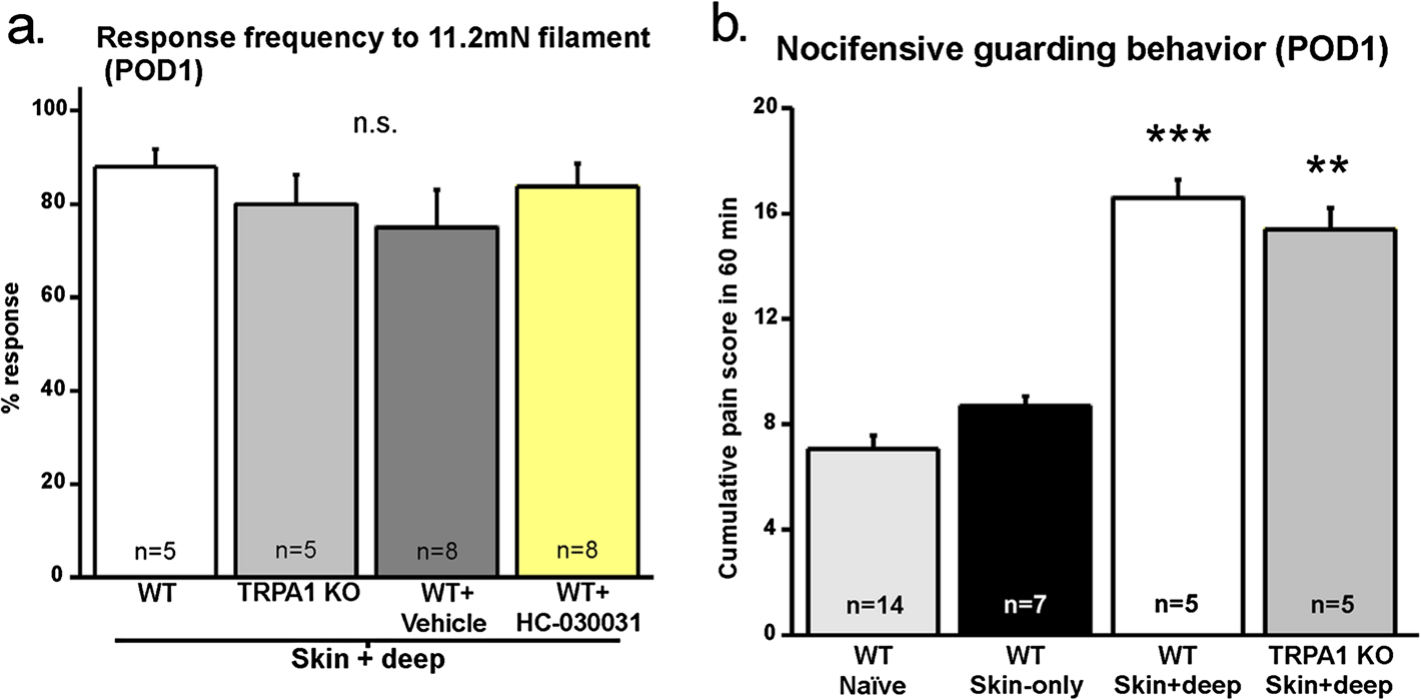
**Mechanical hypersensitivity and guarding behavior is prevalent after skin plus deep incision with genetic knockout and pharmacological inhibition of TRPA1. a**. Response frequency to 11.2mN filament on POD1 for skin plus deep incised WT, TRPA1 KO, WT treated with vehicle and WT treated with TRPA1 antagonist, HC-030031. There is no difference in mechanical hypersensitivity after skin plus deep incision with genetic knockout or pharmacological block of TRPA1. 5 mice per each WT and TRPA1 KO groups; 8 mice per each WT groups treated with either vehicle or HC-030031. **b**. Guarding nocifensive (non-evoked) behavior on POD1 for naïve, skin-only and skin plus deep incised mice. There is no difference in pain score between naïve and skin-only incised mice. Both WT and TRPA1 KO skin plus deep incised mice exhibit increased pain scores, compared to naïve and skin-only incised mice (p < 0.0001; **p < 0.001; applies to comparison to both naïve and skin-only). 14 naïve; 7 WT skin-only incision mice. The skin plus deep incised mice are the same as those used in Figure 2a.

Wei and colleagues [22] found that TRPA1 contributes to mechanical hypersensitivity following skin plus deep incision injury in rats. In order to determine whether deeper tissue injury is required for the contribution of TRPA1 to mechanical hypersensitivity in mice, we conducted skin plus deep incisions on WT and TRPA1 KO mice. We found that the degree of mechanical hypersensitivity following skin plus deep injury was similar to the mechanical hypersensitivity following skin-only incisions (compare incised WT values in Figure 1b POD1 and Figure 2a). There was no difference in mechanical responsiveness between WT and TRPA1 KO mice with skin plus deep incisions (Figure 2a). Next, we determined whether intraplantar HC-030031 would have an effect on mechanical sensitivity following skin plus deep incision. There was no difference in skin plus deep incised mice treated with vehicle and those treated with HC-030031 (Figure 2a). These data suggest that TRPA1 also does not mediate mechanical hypersensitivity following *both* cutaneous and muscle injury in mice. The difference between our results and those from Wei and colleagues [22] may indicate a fundamental difference in pain mechanisms between mouse and rat species, which has been reported by other studies [27-29].

Guarding behavior is thought to be a sign of spontaneous, non-evoked pain [7-10]; therefore, we assessed nocifensive guarding behavior in incised mice by calculating a cumulative pain score over 60 minutes as done by Xu and colleagues [8,9]. Similar to previously reported findings[9], we found that increased guarding behavior is unique to the skin plus deep incision model and is not observed after skin-only incisions as compared to naïve WT mice (Figure 2b). In contrast to the results in rat from Wei and colleagues [22], we found no difference in the guarding behavior between WT and TRPA1 KO mice after skin plus deep incision (Figure 2b). Guarding in both WT and TRPA1 KO mice after skin plus deep incision increased significantly compared to skin-only incised WT mice and there was no difference between these genotypes after skin plus deep incision. Together, our data suggest that TRPA1 does not contribute to mechanical or guarding behavior following cutaneous and muscle injury in mice.

### TRPA1 is not functionally up-regulated in sensory neurons following skin-only incision injury

**Figure 3:**
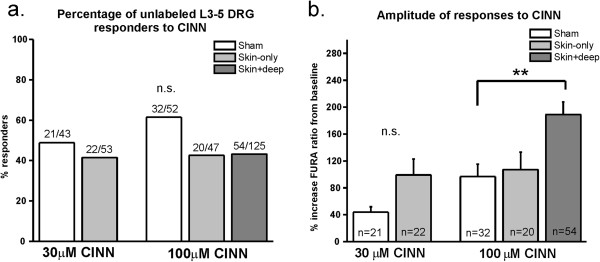
**TRPA1 is not functionally up-regulated in DRG neurons with mixed peripheral targets following skin-only incision injury. a**. Percentage of neurons with mixed peripheral targets responding to cinnamaldehyde (CINN). There is no difference in the percentage of neurons responding to 30 or 100 μM CINN between neurons cultures from sham mice and those from skin-only or skin plus deep incised mice. Neurons were pooled from 6 mice per sham and skin-only incision groups and 4 mice in skin plus deep group. **b**. Amplitude of responses of neurons with mixed peripheral targets to CINN. There is no difference in the amplitude or responses between sham and skin-only neurons to 30 or 100 μM CINN. The amplitude of responses of neurons from skin plus deep incised mice is significantly greater than those from sham and skin-only incised mice (p < 0.0001; **p < 0.01). Same neurons as those in Figure 3a.

In order to determine whether TRPA1 is functionally up-regulated in sensory neurons following skin-only incision injury, we conducted calcium imaging on ipsilateral lumbar 3–5 dorsal root ganglia (DRG) neurons since these DRGs contain the cell bodies of the nerves that terminate in the plantar hind paw region that is incised as well as the region tested during behavior experiments [27]. We isolated and cultured neurons on POD1 since this time point is when we found the greatest behavioral mechanical hypersensitivity (Figure 1a, b). We stimulated neurons with cinnamaldehyde (CINN), a selective agonist for TRPA1 [30,31] and recorded from small-diameter neurons (<27 μm in diameter) since these somata include C fiber-type, TRPA1-expressing neurons [31]. We found no difference in the percentage of responders to 30 or 100 μM CINN between skin-only and sham groups (Figure 3a). There was also no difference in the amplitude of calcium responses to CINN between these groups (Figure 3b). When we recorded neurons from skin plus deep incised mice, we found that the percentage of neurons responding to 100 μM CINN was similar between skin plus deep, skin-only and sham neurons (Figure 3a). Interestingly, the response amplitudes of skin plus deep neurons were significantly greater than those of sham neurons (Figure 3b). These results suggest that TRPA1 is functionally up-regulated in neurons that normally express TRPA1 before injury, after *both* cutaneous and muscle injury in mice. This result does not completely correspond with our behavior data which show that TRPA1 does not contribute to mechanical hypersensitivity or guarding behavior following skin plus deep incision (Figure 2a, b). These disparate results suggest either that TRPA1 plays a minor role in mechanical hypersensitivity behavior following skin plus deep incision below detection levels in our behavior assays, or that TRPA1 plays a role in another pain phenotype following skin plus deep incision which our behavioral assays did not assess.

**Figure 4:**
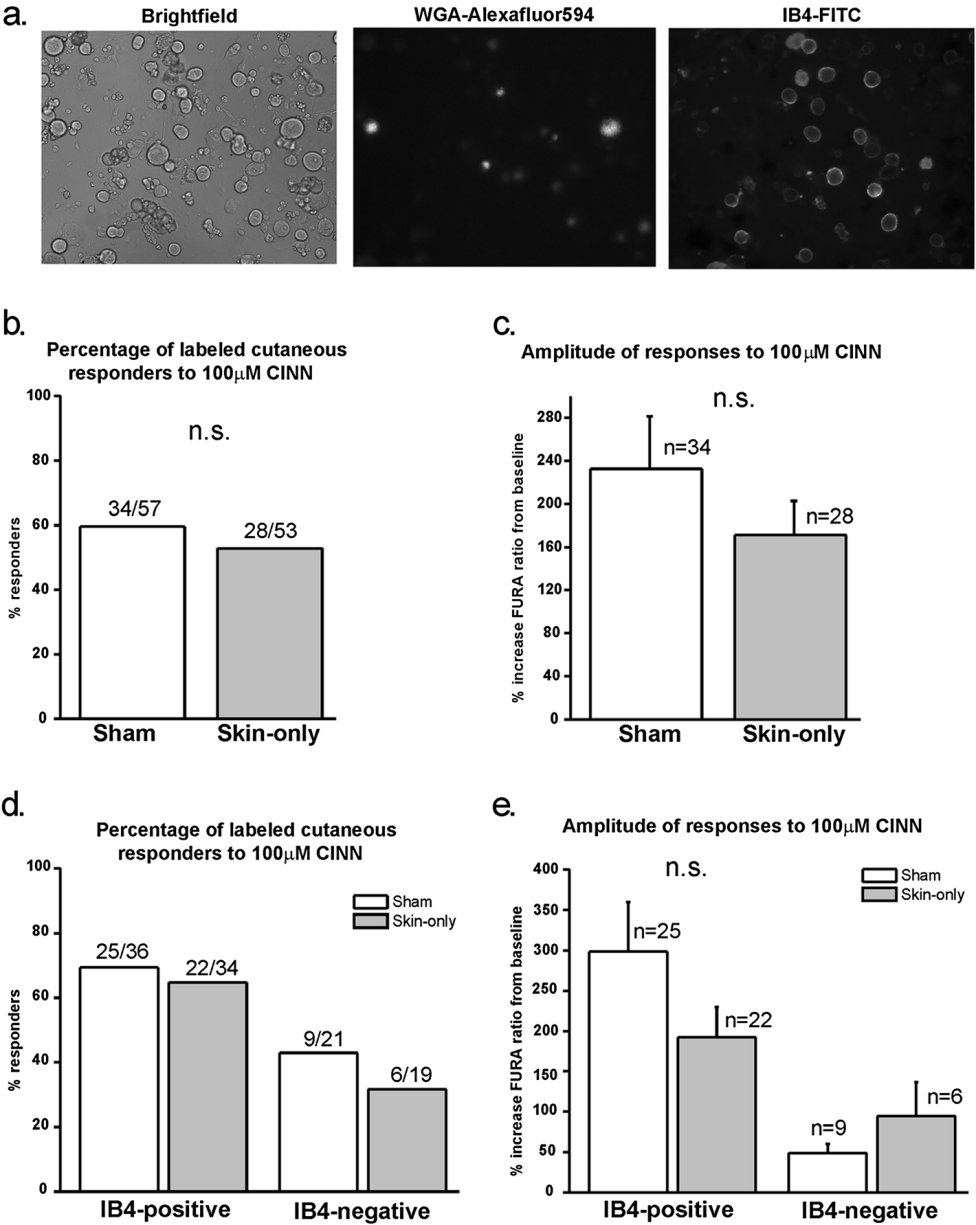
**TRPA1 is not functionally up-regulated in retrogradely labeled cutaneous neurons from glabrous plantar skin following skin-only incision injury. a**. Representative brightfield (left), WGA-Alexafluor594 retrograde label (middle), and IB4-FITC (right) images of ipsilateral lumbar 3–5 neurons from incised mice with retrograde label and IB4 staining (20x objective). Neurons that brightly fluoresced with Alexafluor594 at least 2-times the standard deviation above autofluorescence were considered to be positively stained for the retrograde tracer and recorded. IB4-positive neurons were defined by a halo of FITC labeling around the entire perimeter of the somata of small-diameter (<27 μm) neurons. **b**. Percentage of cutaneous neurons responding to CINN. There is no difference in the percentage of labeled WT neurons responding to 100μM CINN from skin-only incised and sham mice. Neurons were pooled from 3 mice per group. **c**. Amplitude of responses of cutaneous neurons to CINN. Ipsilateral retrograde-labeled neurons from skin-only incised WT mice respond with the same amplitude of intracellular increase to 100μM CINN as neurons from sham mice. Same neurons as those in Figure 4b. **d**. Percentage of cutaneous neurons responding to CINN defined by IB4 staining. There is no difference in percentage of labeled neurons responding to 100μM CINN defined by IB4 binding. Same neurons as those in Figure 4b. **e**. Amplitude of responses of cutaneous neurons responding to CINN defined by IB4 staining. There was no difference in amplitude of responses to 100μM CINN in labeled neurons defined by IB4 staining. Same neurons as those in Figure 4b.

Since neurons from lumbar 3–5 DRGs innervate many anatomical regions and tissue types of the hind limbs [27], the inclusion of neurons that project to regions other than the incision site could dilute any sensitization effect. Therefore, six days prior to skin incision, we injected wheat germ agglutinin conjugated to a fluorophore (WGA-594) into the medial plantar hind paw in order to retrogradely label the somata of neurons that specifically innervate the injured cutaneous region. We recorded from DRG neurons that fluoresced brightly and were at least two standard deviations above autofluorescence, indicating that they took up sufficient tracer and thus projected to the plantar hind paw region (Figure 4a- middle image). Even in this labeled subgroup, we still found no difference in the percentage of responders or amplitude of response to 100 μM CINN in the skin-only incised versus sham groups (Figure 4b, c). Next, we stained these cells with isolectin B4 conjugated to a fluoroscein (IB4). IB4 is used to distinguish between two subsets of small-diameter neurons with distinctive peripheral terminal targets and central projections and are thought to transmit signals for different aspects of pain processing [32-36] (Figure 4a- right image). We did not find a difference in the percentage of responders or a difference in amplitude of responses between sham and skin-only incised IB4-positive neurons or IB4-negative neurons (Figure 4d, e). The results for neurons from sham mice are consistent with those from naïve mice in our previous study [31]. Our data presented here indicate that TRPA1 is not functionally up-regulated in DRG sensory neurons that project to the plantar hind paw cutaneous site after skin-only incision. These results support our findings from behavior experiments which indicate that TRPA1 does not mediate cutaneous incision-induced mechanical hypersensitivity.

### TRPV1 mediates heat hypersensitivity following cutaneous incision injury

**Figure 5:**
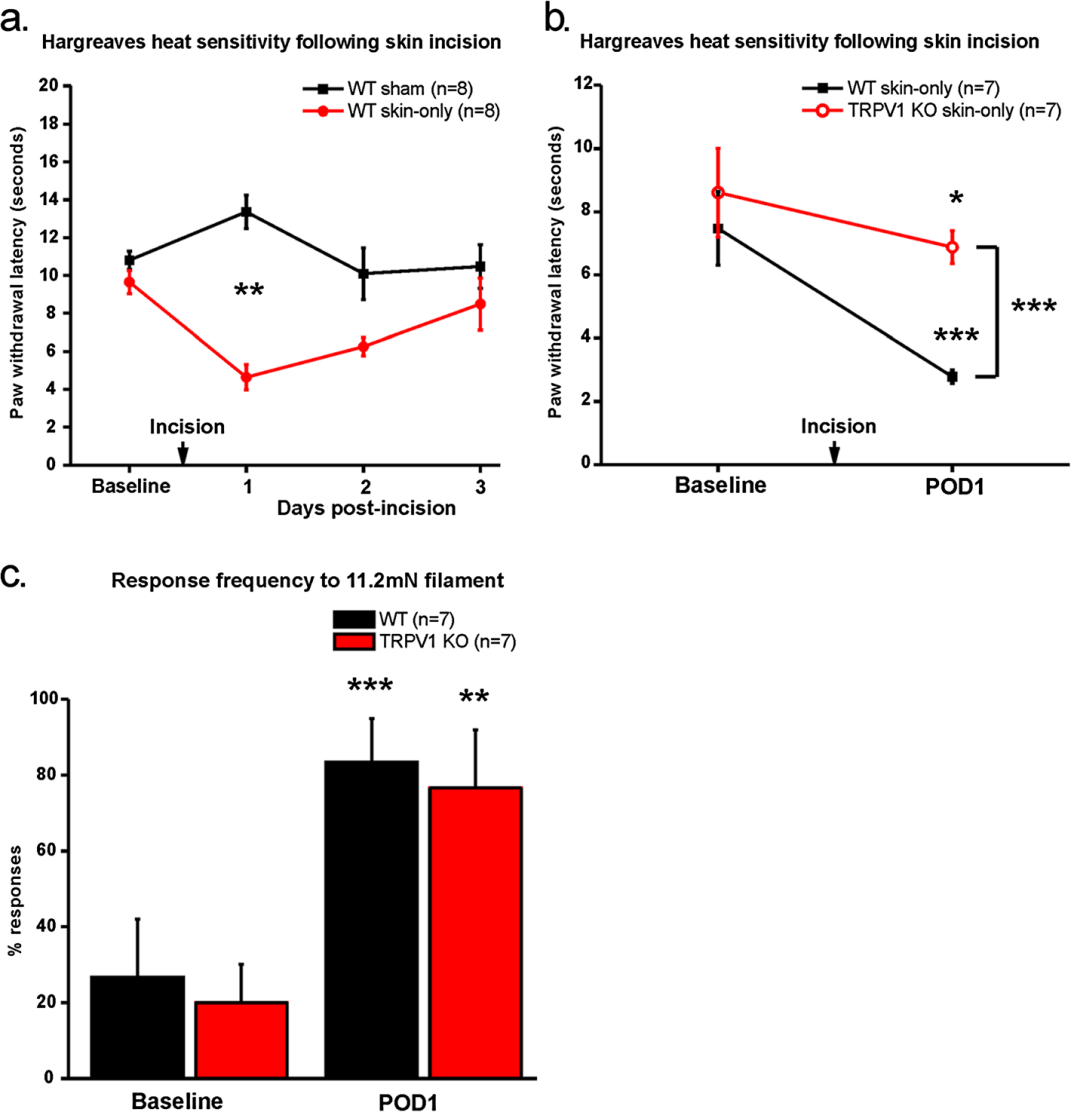
**Skin-only incision induced thermal hypersensitivity is dependent on TRPV1. a**. Heat behavior assay for WT skin-only incised and sham mice. WT mice exhibit decreased paw withdrawal latencies on POD1 after skin-only incision injury as compared to sham controls (p = 0.0013; ***p < 0.0001). 8 mice per group. **b**. Heat behavior assay for WT and TRPV1 KO skin-only incised mice. TRPV1 KO skin-only incised mice exhibit significantly longer paw withdrawal latencies at POD1 than skin incised WT mice (2-way repeated measures ANOVA: p < 0.0001; ***p < 0.0001). TRPV1 mice exhibit an increase in heat sensitivity compared to baseline (paired t-test *p = 0.0022); however, WT mice exhibit a much greater increase in heat sensitivity compared to their baseline values (paired t-test ***p < 0.0001). 7 mice per group. Since multiple statistical tests were conducted, we used assumed p-values <0.025 were significant rather than 0.05. Through nonparametric testing we reached the same conclusion. **c**. Response frequency to 11.2mN filament for WT and TRPV1 KO mice following skin-only incision injury. There was no difference at baseline or POD1 between WT and TRPV1 KO mice (2-way repeated measures ANOVA: p > 0.05; n.s.). Both WT and TRPV1 KO mice exhibit increase mechanical sensitivity on POD1 following skin-only incision injury compared to their respective baseline values (paired t-tests; ***p = 0.0002; **p = 0.0003). Same mice as those in Figure 5b. Since multiple statistical tests were conducted, we assumed p-values <0.025 were significant rather than 0.05. Through nonparametric testing we reached the same conclusion.

Other groups have shown that primary sensory afferent terminals are sensitized to heat following skin plus deep tissue incision injury and that this thermal sensitization is mediated by TRPV1 [10,13-15]. However, it is not known whether the sensitization is due to injury of cutaneous or deep tissue. Thus, we first determined whether skin-only incision leads to heat hypersensitivity. We conducted the Hargreaves thermal behavior assay [37] on sham and skin-only incised WT mice. We found that WT mice exhibited marked heat hypersensitivity following skin-only incision on POD1 and that the heat sensitivity returned to levels similar to baseline by POD3 (Figure 5a). In order to determine whether TRPV1 mediates the heat hypersensitivity, we conducted the heat behavior assay on WT and TRPV1-deficient (TRPV1 KO) [38] mice with skin-only incisions (Figure 5b). We found that both WT and TRPV1 KO mice become significantly more sensitive to heat compared to their baseline values. However, the heat sensitivity of the incised TRPV1 KO mice on POD1 was significantly less than that of the incised WT mice. As expected, we did not observe any effect of TRPV1 on mechanical hypersensitivity following skin-only incision (Figure 5c). These data suggest that although TRPV1 may not be the only contributor to heat hypersensitivity, TRPV1 is a major mediator of the behavioral heat hypersensitivity following cutaneous surgical incision injury.

### TRPV1 is functionally up-regulated in IB4-positive neurons following cutaneous incision injury

**Figure 6:**
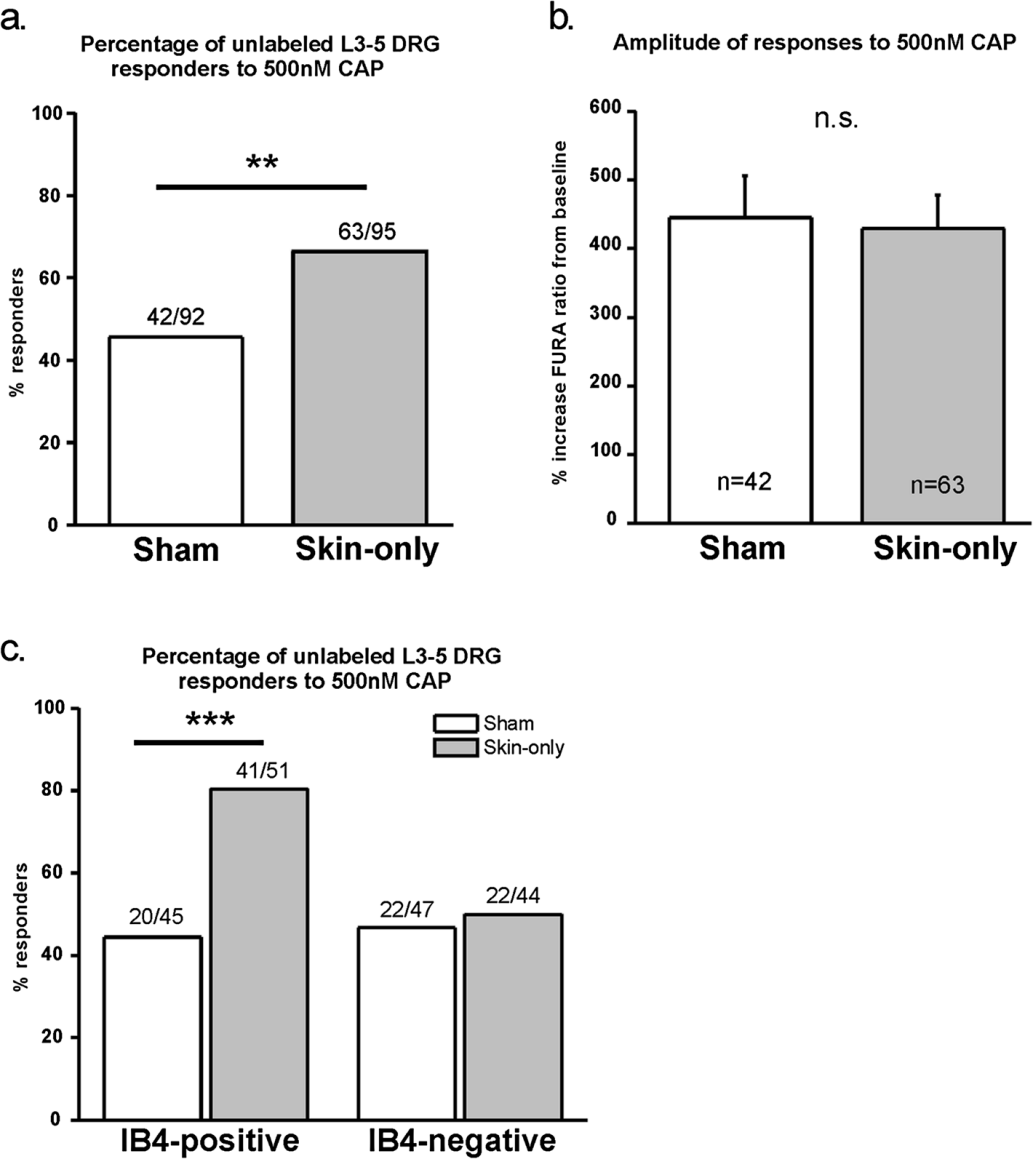
**TRPV1 is functionally up-regulated in IB4-positive, DRG neurons with mixed peripheral targets following skin incision injury. a**. Percentage of neurons with mixed peripheral targets responding to capsaicin (CAP). There are significantly more responders to 500nM CAP among ipsilateral lumber 3–5 neurons from skin-only incised WT mice than from sham mice (**p = 0.0052). Neurons pooled from 3 mice per group. **b**. Amplitude of responses of neurons with mixed peripheral targets to CAP. There is no difference in the amplitude of responses to 500nM CAP between neurons from skin-only incised and sham mice. Same neurons as in Figure 6a. **c**. Percentage of neurons with mixed peripheral targets responding to CAP defined by IB4 binding. Significantly more IB4-positive neurons respond to 500nM CAP from skin-only incised mice than sham mice (overall effect: p = 0.0007; ***p = 0.0003). There is no difference in CAP responsiveness in IB4-negative neurons from incised and sham mice. Same neurons as in Figure 6a.

While we did not find differences in functional TRPA1 in DRG somata after skin incision injury, one possibility might be that functional changes in TRP channels may not occur at the DRG level of the sensory neuron. Therefore, we asked whether sensitization to capsaicin (CAP), a specific agonist of TRPV1, could be observed at the DRG somata level following skin-only incision using the same protocol that we previously used to study TRPA1. As with the other calcium imaging experiments, we isolated neurons on POD1 when the greatest behavioral heat sensitivity occurred (Figure 5a). Small-diameter ipsilateral lumbar 3–5 DRG sensory neurons from skin-only incised mice were significantly more responsive to 500 nM CAP compared to neurons from sham mice (Figure 6a). There was no difference in the amplitude of responses between skin-only and sham neurons (Figure 6b). Since we found an increase in overall percentage of small-diameter neurons responding to CAP, we stratified the neurons by IB4 staining (Figure 4a- right image). We found that the skin incision-induced increase in functional TRPV1 occurred selectively in the IB4-positive population of small-diameter neurons whereas there was no change in the IB4-negative population (Figure 6c).

**Figure 7:**
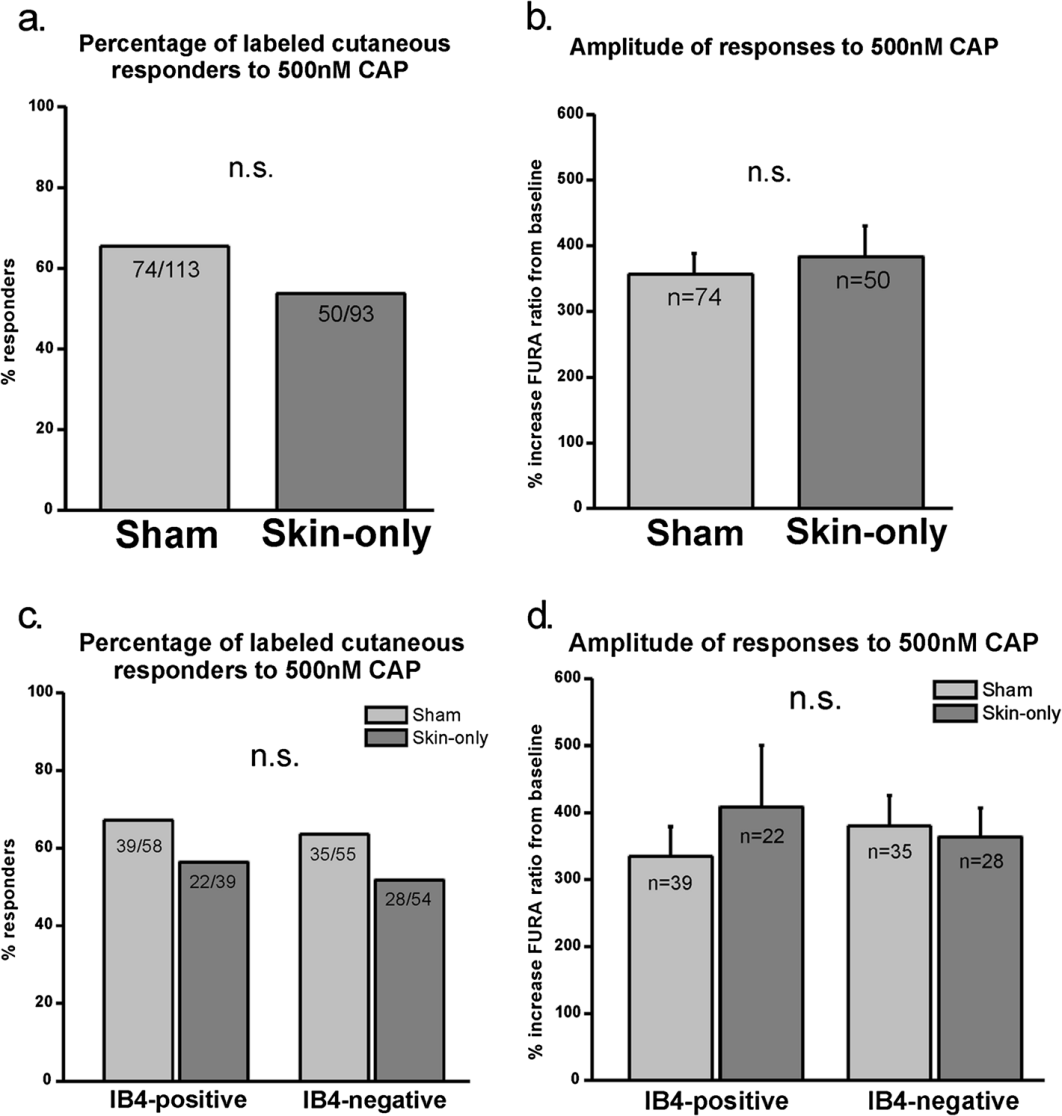
**TRPV1 is not functionally up-regulated in retrogradely labeled cutaneous neurons following skin-only incision injury. a**. Percentage of cutaneous neurons responding to CAP. There is no difference in the percentage of labeled WT neurons responding to 500nM CAP from skin-only incised and sham mice. Neurons were pooled from 9 sham and 8 incised mice; three independent experiments were performed with no differences between experiments. **b**. Amplitude of responses of cutaneous neurons to CAP. The amplitude of responses to 500nM CAP for neurons from skin-only incised mice is not different than those from sham mice. Same neurons as in Figure 7a. **c**. Percentage of cutaneous neurons responding to CAP defined by IB4 staining. There is no difference in percentage of labeled neurons responding to 500nM CAP defined by IB4 binding. Same neurons as in Figure 7a. **d**. Amplitude of responses of cutaneous neurons responding to CAP defined by IB4 staining. The amplitude of responses to 500nM CAP for IB4-positive, labeled neurons from skin-only incised mice is not different than those from sham mice. Likewise, there is no difference in CAP responsiveness in IB4-negative neurons from incised and sham mice. Same neurons as in Figure 7a.

We then conducted retrograde labeling with WGA-594 injected into the medial plantar hind paw in order to identify the somata of plantar skin-projecting afferents, as we did for experiments with TRPA1 (Figure 4a- middle image). The same criteria used in the calcium imaging experiments for TRPA1 for selecting labeled neurons were applied to these experiments. Interestingly, we did not find an increase in the percentage of small-diameter neurons responding to CAP following skin-only incision (Figure 7a) as we had found in mixed-target, unlabeled neurons (Figure 6a). There was also no change in response amplitude with skin-only incision compared to sham controls (Figure 7b). When we stratified the small-diameter populations with IB4 staining there was no difference in the percentage (Figure 7c) or response amplitude (Figure 7d) of either IB4-positive or –negative neurons responding to CAP between sham and skin incised mice. Since we sampled approximately 100 neurons from 8–9 mice (8–10 neurons per mouse), it is unlikely that we missed detecting sensitization due to a low sample size. Together, these data indicate that although we observed functional up-regulation of TRPV1 among somata with mixed peripheral target tissues, we did not detect functional changes of TRPV1 among cutaneous neurons that specifically innervate the plantar glabrous skin incision site. This suggests that perhaps the contribution to heat hypersensitivity is due to TRPV1 functional up-regulation among nerves that terminate in tissues adjacent or deep to the cutaneous surgical incision.

## Discussion

Here we set out to determine the contribution of TRPA1 to mechanical hypersensitivity following skin-only incision injury in mice. Our results demonstrate that skin incision induces mechanical hypersensitivity in TRPA1-deficient “knockout” (KO) mice similar to levels measured in wild-type mice. Additionally, we observed no effect of the TRPA1 antagonist HC-030031 on mechanical hypersensitivity following skin injury in wild-type mice. Further, we found no functional up-regulation of TRPA1 in lumbar DRGs from wild-type mice after skin incision injury using calcium imaging. When we recorded from neurons that specifically projected to the plantar region of the ipsilateral hind paw where the skin incision was performed, there was also no functional up-regulation of TRPA1 at the DRG level. Together, these findings indicate that TRPA1 does not mediate mechanical hypersensitivity following cutaneous surgical incision injury in mice.

Several studies have shown that TRPV1 mediates heat hypersensitivity following skin plus deep incision [13-15]. Therefore, we investigated the role of TRPV1 in heat hypersensitivity with cutaneous-only injury. We found that TRPV1 KO mice exhibit a modest increase in heat sensitivity following skin incision injury; however, incised TRPV1 knockout mice exhibit significantly less heat hypersensitivity compared to wild-type mice. Since we observed a small, yet significant, increase in heat sensitivity in TRPV1 KO mice following skin incision, other receptors likely play a role in the heat sensitivity. Other contributors to heat hypersensitivity may include TRPV3 and TRPV4 [39-43] as well as the recently identified sensory neuron heat channels TRPM3 [44] and Anoctamin 1 (ANO1) [45]. Nonetheless, our data indicate that TRPV1 is the major contributor to behavioral heat hypersensitivity following cutaneous incision injury.

Beyond behavioral assays, we show for the first time that TRPV1 is functionally up-regulated in isolated DRG sensory neurons following skin incision injury. The behavioral heat hypersensitivity present after skin incision is likely mediated, at least in part, by this functional up-regulation of TRPV1 which may be attributed to increased TRPV1 protein or mRNA expression, increased receptor translocation to the plasma membrane, or modulation of existing receptors on sensory neurons. Further experimentation is necessary to determine the exact mechanism of TRPV1 functional up-regulation. Interestingly, our data demonstrate that functional expression of TRPV1 increases specifically in IB4-positive neurons, which are a subset of small-diameter neurons that have been shown to terminate more superficially in skin, centrally project to the inner lamina II of the spinal cord, and transmit pain signals into central processing centers that influence the affective components of pain [32-36]. TRPV1 has been shown to mediate heat hypersensitivity in other pain models such as inflammation and nerve injury, and studies have shown that these models induce increased TRPV1 expression in IB4-positive sensory neurons [38,46,47]. Accumulating evidence suggests that IB4-positive C fiber-type neurons are the most malleable subset of sensory neurons to sensitization after tissue injury [38,47], and evidence from Levine and colleagues strongly suggests that hyperalgesic priming occurs selectively in the IB4-binding population following injury [48,49]. Therefore, our data suggest that the mechanism behind heat hypersensitivity in postoperative pain may be largely mediated by cutaneous incision injury-driven TRPV1 functional up-regulation in IB4-positive C fiber-type neurons.

Although we found that skin incision induced functional up-regulation of TRPV1 among non-labeled L3-5 DRG neurons that project to mixed peripheral target tissues, we failed to detect functional up-regulation of TRPV1 among neurons that specifically innervated the injured glabrous skin. Spofford and Brennan [50] reported tissue-specific expression of growth factors following skin plus deep incision injury, which may explain the disparity in our results. Nerve growth factor (NGF) was found to be over-expressed in skin from 4hr to 10 days following incision [50], and this growth factor has been previously shown to potentiate functional TRPV1 preferentially in muscle afferent neurons but far less so in cutaneous neurons [51]. Up-regulation of NGF in incised skin may drive the functional up-regulation of TRPV1 among neurons with mixed peripheral target tissues, which include neurons that terminate in muscle. Down-regulation of other growth factors, including artemin, was previously reported in skin at 24hr post incision [50]. Artemin has been previously shown to induce TRPV1 sensitization primarily among cutaneous afferents [51,52]. The down-regulation of artemin and other growth factors that primarily sensitize cutaneous neurons could explain why we did not observe functional up-regulation of TRPV1 among glabrous skin-specific neurons following skin-only incision. However, a number of studies have shown that cutaneous afferent nerves *are* sensitized to heat following skin plus deep incision injury and that the sensitization to heat in cutaneous afferents is mediated by TRPV1 [10,12,14,15]. When growth factor expression was evaluated in incised *muscle* tissue, the expression of artemin was up-regulated at 24hr post skin plus deep incision [50]. Up-regulation of artemin in injured muscle tissue could possibly sensitize TRPV1 in cutaneous neurons. Taken together, we have found that cutaneous incision injury drives increased functional expression of TRPV1 among neurons with mixed peripheral targets which includes muscle afferents but not among neurons that terminate within the superficial region of glabrous skin injury. These findings along with previous studies may suggest that deep incision injury is required to sensitize TRPV1 among glabrous skin afferents.

## Conclusions

Our findings indicate that TRPA1 does not mediate the mechanical hypersensitivity following cutaneous surgical incision injury, although it may be a component of pain following deep tissue injury that includes fascia and muscle. On the other hand, we observed functional up-regulation of TRPV1 in IB4-binding DRG C fiber-type sensory neurons which may be a key underlying mechanism driving the heat hypersensitivity following skin surgical incision injury.

## Methods

### Materials

Cinnamaldehyde (CINN) and capsaicin (CAP) were purchased from Sigma. The same lot was used throughout all experiments. Cells were superfused for 3 min with CINN or 1 min with CAP.

### Animals

Experiments were conducted on 4–6 month old C57BL/6J (WT) male mice (Jackson Laboratories) and male TRPA1^-/-^ (TRPA1 KO) mice in which the exons essential for the *Trpa1* gene function were deleted [23]. The TRPA1 KO mouse line was created on a C57BL/6J background. Male TRPV1^-/-^ mice (TRPV1 KO; B6.129S4-TRPV1tm1^Jul^; Jackson Laboratories) [24] were 2–4 months old and tested with age-matched male C57BL/6J (WT) mice. All experimental procedures have been approved by the Institutional Animal Care and Use Committee of the Medical College of Wisconsin.

### Plantar incision

Anesthesia in mice was induced with 5% isoflurane at 2 L/min delivered into a sealed induction chamber and maintained with 1.5% isoflurane at 500 mL/min with a nose cone. The plantar hind paw was swabbed with Betadine and 70% ethanol. As described previously [6] and adapted from the rat model of postoperative pain [7], a 5-mm longitudinal incision starting 2-mm from the proximal edge of the heel and extending towards the toes was made through the glabrous skin using a sterile number-11 scalpel blade. Unless noted, no further tissue except skin was incised. To evaluate deep tissue incision injury pain in a subset of mice, the underlying muscle was elevated with sterile forceps and incised longitudinally, leaving the muscle origin and insertion intact [6]. The skin was then apposed with 2 sutures of 5–0 nylon on a 3/8 reverse cutting needle. Mice recovered from anesthesia in a plastic bottom cage placed on a heating pad. Sutures were maintained for the duration of the experiments. Mice in which both sutures were chewed out before postoperative day 2 (POD2) were removed from the study. Sham mice were anesthetized for the same average duration and with the same dose as the incised mice but not incised or sutured. The experimenter was blinded to genotype during surgeries.

### Mechanical frequency behavior test

Withdrawal responses to punctuate mechanical stimulation were determined using calibrated Von Frey filaments (EbInstruments). Mice were acclimated for 1 hour within a floor-less Plexiglas chamber placed upon a metal mesh platform. Monofilaments were applied to the ipsilateral plantar paw within 1-mm of the incision for a total of 10 applications, beginning with the lowest force monofilament. The percentage of responses out of the total applications was used as the score (% response) per animal. A greater percentage of responses after incision injury as compared to sham values was considered an indication of mechanical hypersensitivity. The investigator was blinded to genotype during all behavioral testing in the study.

### Hargreaves heat sensitivity behavior test

Heat sensitivity was assessed using a radiant heat source for Hargreaves heat and tail flick assays (IITC, Life Sciences Instruments). Mice were acclimated for 1 hour in Plexiglas chambers placed upon a glass platform. Paw withdrawal latencies were assessed by applying a focused radiant heat source underneath the glass floor to the ipsilateral paw within 1-mm of the incision for a total of 4 applications. There was an automatic cut-off at 20 seconds to avoid tissue injury. The heat source was applied to the same plantar hind paw region on the sham mice. Withdrawal latencies were averaged to a single value per animal. A reduction in withdrawal latency after incision injury as compared to baseline or sham values was considered an indication of heat hypersensitivity.

### Guarding behavior

Guarding behavior was assessed as previously described [8,9]. Mice were placed in Plexiglas chambers placed upon a metal mesh platform. Ipsilateral hind paws were observed during a 1-minute period repeated every 5 minutes for 1 hour. Hind paws were scored (0, 1 or 2) depending on the position of the paw on the mesh. Zero was scored when the incised plantar area was in contact with the mesh. A score of 1 was given if the paw region touched the mesh but did not distort or blanch the incised region, and a 2 was scored when the hind paw was completely off the mesh. The corresponding area of the plantar hind paw was used to score sham and naïve mice. The sum of the scores during the 60-min testing period was the total score for the animal.

### HC-030031 intraplantar injection

Mice were briefly anesthetized with isoflurane. The ipsilateral plantar hind paw was injected subcutaneously with 30 μl HC-030031 (100 μg/site, Sigma) or vehicle (0.5% DMSO and 0.25% Tween-80 in PBS). The mice were then acclimated to the behavioral apparatus for 1 hour to allow for maximal drug effect before behavior testing [25]. The experimenter was blinded to chemical treatment.

### Retrograde labeling of cutaneous neurons

In mice, one medial plantar hind paw was injected with 20 μl 1% wheat germ agglutinin conjugated to Alexafluor594 (WGA-594) in sterile saline (Invitrogen). Our prior studies have shown that bright fluorescence is visible in isolated somata by 6 days after plantar paw injection with WGA-594 [31]. Six days after injection, the injected hind paw underwent sham or skin incision procedures. The next day, ipsilateral lumbar 3–5 DRG neurons were cultured as described below. Cells that fluoresced clearly above background fluorescence levels (≥ 2 standard deviations above autofluorescence) were targeted for calcium imaging experiments (Figure 3a- middle image).

### DRG culture

Prior to DRG dissection, mice were briefly anesthetized with isoflurane via inhalation (Midwest Veterinary Supply) and euthanized by decapitation. Lumbar dorsal root ganglia (DRG) 3–5 were excised ipsilateral to the skin-only incision and on the same side in sham animals and placed into 1 ml Hank’s Balanced Salt Solution (Gibco). After DRG extraction, 1 ml HBSS was replaced with Dulbecco’s Modified Eagle’s Medium/Ham’s nutrient mixture F-12 (DMEM/Hams-F12; Gibco). The ganglia were incubated at 37°C and 5% CO_2_ with 1 mg/ml collagenase Type IV (Sigma), followed by incubation with 0.05% trypsin (Sigma) for 40 and 45 min, respectively. The ganglia were washed and resuspended in complete cell medium, then dissociated into single neurons via trituration. The neurons were plated onto laminin-coated glass coverslips and incubated for 2 hours at 37°C and 5% CO_2_ to allow adherence. Following incubation, complete cell medium was added to flood each well. Complete cell medium consisted of DMEM/Hams-F12, 10% heat-inactivated horse serum, 2mM L-glutamine, 0.8% D-glucose, 100 units penicillin and 100ug/ml streptomycin and no exogenous growth factors were added. Calcium imaging experiments were performed 18-24hr after cells were plated.

### Calcium imaging and analysis

Calcium imaging was performed using dual-wavelength fluorescent calcium indicator FURA-2AM (Invitrogen). Cells cultured and plated as described above were loaded with 2.5 μg/ml FURA-2AM in extracellular buffer (in mM: 150 NaCl, 10 HEPES, 8 glucose, 5.6 KCl, 2 CaCl_2_ and 1 MgCl_2_; pH 7.4, 320 ± 3 mOsm) containing 2% BSA for 45 min at room temperature, followed by a 30 min wash period for de-esterification. Coverslips with loaded cells were mounted onto a perfusion chamber and superfused with buffer at a constant rate of 6 ml/min. Fluorescence images were captured with a cooled CCD camera (CoolSNAP; Photometrics, Tucson, AZ). Metafluor imaging software was utilized in order to detect and analyze intracellular calcium changes throughout the experiment (Molecular Devices, Sunnyvale, CA). A neuron exhibiting ≥20% intracellular calcium increase from baseline was considered a responder. All neurons were tested with only one concentration of one agonist. At the end of each protocol, 50mM KCl extracellular solution was used to depolarize neurons, thereby allowing for identification of viable neurons from non-neuronal cells or non-functioning neurons. Neurons were considered small-diameter if the average diameter of the longest and shortest axis was less than 27 μm.

### IB4 staining

Upon completion of imaging protocol, neurons were incubated with 10 μg/ml isolectin B4 (IB4) conjugated to fluorescein isothiocyanate (FITC; Sigma) for 10 min and then washed with extracellular buffer for 1 min. IB4-positive neurons were identified as those that retained a complete halo of FITC stain around the perimeter of the soma after washing (Figure 3a- right image).

### Statistics

Statistics were conducted using GraphPad Instat (version 3.06, Graphpad Software, Inc) or GraphPad Prism (version 5.04, Graphpad Software, Inc). Behavior data were compared using repeated measures 2-way ANOVA with Bonferroni post-hoc tests or paired t-tests. Calcium imaging data for percentage of responders were analyzed using Chi-square and Fisher’s exact test, and data for amplitude of responses were compared using unpaired t-tests or 2-way ANOVA tests with Tukey post-hoc tests. Error bars on graphs represent the standard error of the mean. P-values <0.05 were considered significant unless noted.

## Abbreviations

TRPV1: Transient receptor potential vanilloid 1; TRPA1: Transient receptor potential ankyrin 1; DRG: Dorsal root ganglion; WT: Wild type; IB4: Isolectin B4; TRPA1 KO: Transient receptor potential ankyrin 1 knockout; TRPV1 KO: Transient receptor potential vanilloid 1 knockout; POD: Postoperative day; CINN: Cinnamaldehyde; WGA: Wheat germ agglutinin; CAP: Capsaicin.

## Competing interests

The authors declare that they have no competing interests.

## Authors’ contributions

MEB contributed to the conception and design of the study, acquisition of the data, analysis and interpretation of the data, drafted and revised manuscript. CLS contributed to conception and design of the study, interpretation of the data, writing and revising the manuscript. Both authors read and approved the final manuscript.

## References

[B1] Apfelbaum JL Chen C Mehta SS Gan TJ Postoperative pain experience: results from a national survey suggest postoperative pain continues to be undermanaged Anesth Analg 2003 97 534 540 table 10.1213/01.ANE.0000068822.10113.9E 12873949

[B2] Guignard B Bossard AE Coste C Sessler DI Lebrault C Alfonsi P Fletcher D Chauvin M Acute opioid tolerance: intraoperative remifentanil increases postoperative pain and morphine requirement Anesthesiology 2000 93 409 417 10.1097/00000542-200008000-00019 10910490

[B3] Gear RW Miaskowski C Gordon NC Paul SM Heller PH Levine JD The kappa opioid nalbuphine produces gender- and dose-dependent analgesia and antianalgesia in patients with postoperative pain Pain 1999 83 339 345 10.1016/S0304-3959(99)00119-0 10534607

[B4] Bennett DL Dmietrieva N Priestley JV Clary D McMahon SB TrkA, CGRP and IB4 expression in retrogradely labelled cutaneous and visceral primary sensory neurones in the rat Neurosci Lett 1996 206 33 36 10.1016/0304-3940(96)12418-6 8848275

[B5] Perry MJ Lawson SN Differences in expression of oligosaccharides, neuropeptides, carbonic anhydrase and neurofilament in rat primary afferent neurons retrogradely labelled via skin, muscle or visceral nerves Neuroscience 1998 85 293 310 10.1016/S0306-4522(97)00629-5 9607720

[B6] Pogatzki EM Raja SN A mouse model of incisional pain Anesthesiology 2003 99 1023 1027 10.1097/00000542-200310000-00041 14508341

[B7] Brennan TJ Vandermeulen EP Gebhart GF Characterization of a rat model of incisional pain Pain 1996 64 493 501 10.1016/0304-3959(95)01441-1 8783314

[B8] Xu J Brennan TJ Comparison of skin incision vs. skin plus deep tissue incision on ongoing pain and spontaneous activity in dorsal horn neurons Pain 2009 144 329 339 10.1016/j.pain.2009.05.019 19527922 PMC2759309

[B9] Xu J Brennan TJ Guarding pain and spontaneous activity of nociceptors after skin versus skin plus deep tissue incision Anesthesiology 2010 112 153 164 10.1097/ALN.0b013e3181c2952e 19996955 PMC2907154

[B10] Banik RK Brennan TJ Spontaneous discharge and increased heat sensitivity of rat C-fiber nociceptors are present in vitro after plantar incision Pain 2004 112 204 213 10.1016/j.pain.2004.08.026 15494202

[B11] Kang S Brennan TJ Chemosensitivity and mechanosensitivity of nociceptors from incised rat hindpaw skin Anesthesiology 2009 111 155 164 10.1097/ALN.0b013e3181a16443 19512876 PMC2737702

[B12] Banik RK Brennan TJ Sensitization of primary afferents to mechanical and heat stimuli after incision in a novel in vitro mouse glabrous skin-nerve preparation Pain 2008 138 380 391 10.1016/j.pain.2008.01.017 18316159 PMC3787122

[B13] Pogatzki-Zahn EM Shimizu I Caterina M Raja SN Heat hyperalgesia after incision requires TRPV1 and is distinct from pure inflammatory pain Pain 2005 115 296 307 10.1016/j.pain.2005.03.010 15911156

[B14] Kang S Wu C Banik RK Brennan TJ Effect of capsaicin treatment on nociceptors in rat glabrous skin one day after plantar incision Pain 2010 148 128 140 10.1016/j.pain.2009.10.031 19948377 PMC2815239

[B15] Banik RK Brennan TJ Trpv1 mediates spontaneous firing and heat sensitization of cutaneous primary afferents after plantar incision Pain 2009 141 41 51 10.1016/j.pain.2008.10.004 19010598 PMC2654272

[B16] Ji G Zhou S Carlton SM Intact Adelta-fibers up-regulate transient receptor potential A1 and contribute to cold hypersensitivity in neuropathic rats Neuroscience 2008 154 1054 1066 10.1016/j.neuroscience.2008.04.039 18514429 PMC2530901

[B17] McGaraughty S Chu KL Perner RJ Didomenico S Kort ME Kym PR TRPA1 modulation of spontaneous and mechanically evoked firing of spinal neurons in uninjured, osteoarthritic, and inflamed rats Mol Pain 2010 6 14 10.1186/1744-8069-6-14 20205719 PMC2841076

[B18] Kobayashi K Fukuoka T Obata K Yamanaka H Dai Y Tokunaga A Noguchi K Distinct expression of TRPM8, TRPA1, and TRPV1 mRNAs in rat primary afferent neurons with adelta/c-fibers and colocalization with trk receptors J Comp Neurol 2005 493 596 606 10.1002/cne.20794 16304633

[B19] Caspani O Zurborg S Labuz D Heppenstall PA The contribution of TRPM8 and TRPA1 channels to cold allodynia and neuropathic pain PLoS One 2009 4 e7383 10.1371/journal.pone.0007383 19812688 PMC2753652

[B20] Atoyan R Shander D Botchkareva NV Non-neuronal expression of transient receptor potential type A1 (TRPA1) in human skin J Invest Dermatol 2009 129 2312 2315 10.1038/jid.2009.58 19282836

[B21] Lennertz RC Kossyreva EA Smith AK Stucky CL TRPA1 mediates mechanical sensitization in nociceptors during inflammation PLoS One 2012 7 e43597 10.1371/journal.pone.0043597 22927999 PMC3426543

[B22] Wei H Karimaa M Korjamo T Koivisto A Pertovaara A Transient receptor potential ankyrin 1 ion channel contributes to guarding pain and mechanical hypersensitivity in a rat model of postoperative pain Anesthesiology 2012 117 137 148 10.1097/ALN.0b013e31825adb0e 22588108

[B23] Kwan KY Allchorne AJ Vollrath MA Christensen AP Zhang DS Woolf CJ Corey DP TRPA1 contributes to cold, mechanical, and chemical nociception but is not essential for hair-cell transduction Neuron 2006 50 277 289 10.1016/j.neuron.2006.03.042 16630838

[B24] Caterina MJ Leffler A Malmberg AB Martin WJ Trafton J Petersen-Zeitz KR Koltzenburg M Basbaum AI Julius D Impaired nociception and pain sensation in mice lacking the capsaicin receptor Science 2000 288 306 313 10.1126/science.288.5464.306 10764638

[B25] da Costa DS Meotti FC Andrade EL Leal PC Motta EM Calixto JB The involvement of the transient receptor potential A1 (TRPA1) in the maintenance of mechanical and cold hyperalgesia in persistent inflammation Pain 2010 148 431 437 10.1016/j.pain.2009.12.002 20056530

[B26] Mansikka H Zhao C Sheth RN Sora I Uhl G Raja SN Nerve injury induces a tonic bilateral mu-opioid receptor-mediated inhibitory effect on mechanical allodynia in mice Anesthesiology 2004 100 912 921 10.1097/00000542-200404000-00022 15087627

[B27] Rigaud M Gemes G Barabas ME Chernoff DI Abram SE Stucky CL Hogan QH Species and strain differences in rodent sciatic nerve anatomy: implications for studies of neuropathic pain Pain 2008 136 188 201 10.1016/j.pain.2008.01.016 18316160 PMC2700063

[B28] Price TJ Flores CM Critical evaluation of the colocalization between calcitonin gene-related peptide, substance P, transient receptor potential vanilloid subfamily type 1 immunoreactivities, and isolectin B4 binding in primary afferent neurons of the rat and mouse J Pain 2007 8 263 272 17113352 10.1016/j.jpain.2006.09.005 PMC1899162

[B29] Obata K Katsura H Mizushima T Yamanaka H Kobayashi K Dai Y Fukuoka T Tokunaga A Tominaga M Noguchi K TRPA1 induced in sensory neurons contributes to cold hyperalgesia after inflammation and nerve injury J Clin Invest 2005 115 2393 2401 10.1172/JCI25437 16110328 PMC1187934

[B30] Bandell M Story GM Hwang SW Viswanath V Eid SR Petrus MJ Earley TJ Patapoutian A Noxious cold ion channel TRPA1 is activated by pungent compounds and bradykinin Neuron 2004 41 849 857 10.1016/S0896-6273(04)00150-3 15046718

[B31] Barabas ME Kossyreva EA Stucky CL TRPA1 is functionally expressed primarily by IB4-binding, non-peptidergic mouse and rat sensory neurons PLoS One 2012 7 e47988 10.1371/journal.pone.0047988 23133534 PMC3485059

[B32] Dirajlal S Pauers LE Stucky CL Differential response properties of IB(4)-positive and -negative unmyelinated sensory neurons to protons and capsaicin J Neurophysiol 2003 89 513 524 12522198 10.1152/jn.00371.2002

[B33] Fullmer JM Riedl MS Higgins L Elde R Identification of some lectin IB4 binding proteins in rat dorsal root ganglia Neuroreport 2004 15 1705 1709 10.1097/01.wnr.0000136037.54095.64 15257131

[B34] Gerke MB Plenderleith MB Binding sites for the plant lectin bandeiraea simplicifolia I-isolectin B(4) are expressed by nociceptive primary sensory neurones Brain Res 2001 911 101 104 10.1016/S0006-8993(01)02750-0 11489450

[B35] Zylka MJ Nonpeptidergic circuits feel your pain Neuron 2005 47 771 772 10.1016/j.neuron.2005.09.003 16157268

[B36] Braz JM Nassar MA Wood JN Basbaum AI Parallel “pain” pathways arise from subpopulations of primary afferent nociceptor Neuron 2005 47 787 793 10.1016/j.neuron.2005.08.015 16157274

[B37] Yeomans DC Proudfit HK Characterization of the foot withdrawal response to noxious radiant heat in the rat Pain 1994 59 85 94 10.1016/0304-3959(94)90051-5 7854807

[B38] Vilceanu D Honore P Hogan QH Stucky CL Spinal nerve ligation in mouse upregulates TRPV1 heat function in injured IB4-positive nociceptors J Pain 2010 11 588 599 10.1016/j.jpain.2009.09.018 20015699 PMC2879455

[B39] Moqrich A Hwang SW Earley TJ Petrus MJ Murray AN Spencer KS Andahazy M Story GM Patapoutian A Impaired thermosensation in mice lacking TRPV3, a heat and camphor sensor in the skin Science 2005 307 1468 1472 10.1126/science.1108609 15746429

[B40] Guler AD Lee H Iida T Shimizu I Tominaga M Caterina M Heat-evoked activation of the ion channel, TRPV4 J Neurosci 2002 22 6408 6414 12151520 10.1523/JNEUROSCI.22-15-06408.2002 PMC6758176

[B41] Bang S Yoo S Yang TJ Cho H Hwang SW Farnesyl pyrophosphate is a novel pain-producing molecule via specific activation of TRPV3 J Biol Chem 2010 285 19362 19371 10.1074/jbc.M109.087742 20395302 PMC2885216

[B42] Xu H Ramsey IS Kotecha SA Moran MM Chong JA Lawson D Ge P Lilly J Silos-Santiago I Xie Y TRPV3 is a calcium-permeable temperature-sensitive cation channel Nature 2002 418 181 186 10.1038/nature00882 12077604

[B43] Peier AM Reeve AJ Andersson DA Moqrich A Earley TJ Hergarden AC Story GM Colley S Hogenesch JB McIntyre P A heat-sensitive TRP channel expressed in keratinocytes Science 2002 296 2046 2049 10.1126/science.1073140 12016205

[B44] Vriens J Owsianik G Hofmann T Philipp SE Stab J Chen X Benoit M Xue F Janssens A Kerselaers S TRPM3 is a nociceptor channel involved in the detection of noxious heat Neuron 2011 70 482 494 10.1016/j.neuron.2011.02.051 21555074

[B45] Cho H Yang YD Lee J Lee B Kim T Jang Y Back SK Na HS Harfe BD Wang F The calcium-activated chloride channel anoctamin 1 acts as a heat sensor in nociceptive neurons Nat Neurosci 2012 15 1015 1021 10.1038/nn.3111 22634729

[B46] Yu L Yang F Luo H Liu FY Han JS Xing GG Wan Y The role of TRPV1 in different subtypes of dorsal root ganglion neurons in rat chronic inflammatory nociception induced by complete Freund’s adjuvant Mol Pain 2008 4 61 10.1186/1744-8069-4-61 19055783 PMC2628345

[B47] Breese NM George AC Pauers LE Stucky CL Peripheral inflammation selectively increases TRPV1 function in IB4-positive sensory neurons from adult mouse Pain 2005 115 37 49 10.1016/j.pain.2005.02.010 15836968

[B48] Joseph EK Levine JD Hyperalgesic priming is restricted to isolectin B4-positive nociceptors Neuroscience 2010 169 431 435 10.1016/j.neuroscience.2010.04.082 20457222 PMC2903040

[B49] Ferrari LF Bogen O Levine JD Nociceptor subpopulations involved in hyperalgesic priming Neuroscience 2010 165 896 901 10.1016/j.neuroscience.2009.11.029 19931357 PMC2815163

[B50] Spofford CM Brennan TJ Gene expression in skin, muscle, and dorsal root ganglion after plantar incision in the rat Anesthesiology 2012 117 161 172 10.1097/ALN.0b013e31825a2a2b 22617252 PMC3389501

[B51] Malin S Molliver D Christianson JA Schwartz ES Cornuet P Albers KM Davis BM TRPV1 and TRPA1 function and modulation are target tissue dependent J Neurosci 2011 31 10516 10528 10.1523/JNEUROSCI.2992-10.2011 21775597 PMC3180860

[B52] Elitt CM McIlwrath SL Lawson JJ Malin SA Molliver DC Cornuet PK Koerber HR Davis BM Albers KM Artemin over expression in skin enhances expression of TRPV1 and TRPA1 in cutaneous sensory neurons and leads to behavioral sensitivity to heat and cold J Neurosci 2006 26 8578 8587 10.1523/JNEUROSCI.2185-06.2006 16914684 PMC6674358

